# Efficiency and Precision in Orthodontics: A Case Report and Literature Review on the Simplified Lingual Bonding System

**DOI:** 10.7759/cureus.99528

**Published:** 2025-12-18

**Authors:** Subrat Satapathy, Nikita Agarwal, Suryakanta Das

**Affiliations:** 1 Orthodontics and Dentofacial Orthopaedics, Srirama Chandra Bhanja Dental College and Hospital, Cuttack, IND

**Keywords:** anatomical brackets, indirect bonding, lingual orthodontics, simplified lingual bonding system, transfer trays

## Abstract

The laboratory stage of lingual orthodontics imposes specific difficulties unique to the specialty, including anatomical variance, bracket placement discrepancies, and an accurate amount of overcorrection. Traditional bonding methods often require added compensatory resin pads, which can affect the patient's hygiene and bracket retention. The simplified lingual bonding system was developed to address these issues by replacing traditional bonding pads with bracket bases designed according to their anatomy, providing optimal placement within the lingual fossa. This bonding method uses a simplified bonding technique and eliminates the added step of creating a setup model while maintaining a level of accuracy in the placement. The method will include a step-by-step process for proper surface preparation, bracket height in position, and obtaining a custom transfer tray. The enhanced process minimizes the challenges of indirect bonding, along with improved clinical success, and provides the patient with smaller brackets, leading to a smaller appliance that should increase comfort. By integrating standardized protocols with innovative laboratory techniques, the simplified lingual bonding system represents a significant advancement in lingual orthodontics.

## Introduction and background

The popularity of aesthetic orthodontic treatment has contributed to the growing demand for lingual braces, which offer the advantage of being almost invisible while remaining highly effective in treating malocclusions. Although its popularity has been increasing, lingual orthodontics, where brackets are placed on the lingual (tongue-facing) surfaces of teeth, presents unique challenges, including restricted access, limited visibility, and the complex anatomy of the lingual surface of teeth [1]. To overcome these challenges, indirect bonding methods, such as intra-oral scanning, 3D printing [2], computer-aided design (CAD) [3], and other orthodontic software, have revolutionized bonding techniques and tray fabrication, allowing for more precise and efficient bracket positioning than direct bonding. The utilization of 3D printing technology has optimized the production process of transfer trays, thereby enhancing the efficiency of lingual bracket placement. CAD/computer-aided manufacturing (CAM) software facilitates the virtual positioning of brackets on digital models, which is followed by CAM processes to produce highly accurate transfer trays with superior accuracy in mesiodistal, buccolingual, and occlusal-gingival dimensions and rotational alignment. Moreover, developing orthodontic-specific software, such as Ubrackets [4], has facilitated the design of personalized lingual brackets and indirect bonding trays. It has enabled orthodontists to digitally position brackets and produce patient-specific devices customized to individual anatomical differences [4]. This advancement allows practitioners to maintain control over the design and production stages, reducing reliance on external laboratories and enabling faster workflows. Recent evidence has further validated the accuracy of digital workflows, with Jaber et al. demonstrating comparable trueness and precision between intraoral scans and poured digital models for indirect bonding applications, supporting reliable virtual bracket positioning in lingual orthodontics [5].

Although these techniques and advancements have significantly contributed to treating orthodontic conditions, the cost-effectiveness of these methods has been a point of discussion for broader accessibility [6]. One approach uses light-cured adhesives to enhance bond strength and ensure precise bracket placement, reducing positioning errors and bracket failure [7]. This technique involves a two-stage process: brackets are first positioned on a plaster model in the laboratory and then transferred to the patient's mouth using a tray bonded to the etched enamel surface. However, current techniques have issues such as excess adhesive flash around the brackets, which requires removal with a round bur and handpiece [8]. Additionally, these techniques are difficult to use in crowded dentitions, may result in inaccurate curing, pose challenges in tray removal, and are not cost-effective [9]. An umbrella review by Alhafi et al. summarized existing systematic reviews on indirect bonding techniques, concluding that indirect methods generally improve bracket placement accuracy and reduce chairside variability, although technique sensitivity and laboratory complexity remain consistent challenges [10].

This literature review aims to evaluate the efficiency and precision of simplified lingual indirect-bonding systems used in orthodontic laboratory workflows. Specifically, it focuses on contemporary laboratory-based techniques - including CAD/CAM-assisted bracket positioning, digital model utilization, and transfer tray fabrication - while considering reported clinical implications, such as bonding accuracy, chairside efficiency, and technique sensitivity. The review synthesizes evidence from in vitro, in silico, and clinical studies published within the last 15 years, with emphasis on studies addressing digital workflows, indirect bonding accuracy, and tray design modifications. By clarifying laboratory protocols and their clinical relevance, this review seeks to provide an accessible overview for both specialists and clinicians less familiar with indirect bonding systems. This review introduces a simplified bonding system that modifies existing techniques by utilizing the lingual surface's surface topography and inclination of the lingual surface [11,12]. It includes creating incisal and occlusal windows to improve access for cleaning excess adhesive and enhance the curing process by allowing light to reach the adhesive [13]. These windows also facilitate tray removal and reduce bulk, making the tray easier to position. This refined bonding process improves efficiency, accuracy, and patient adherence in lingual orthodontics [13].

While an extension of conventional methods, the simplified technique introduces new laboratory procedures that, combined with the ease of bonding concepts, facilitate the development of an innovative process in lingual orthodontics. However, the anatomical design of the bracket base and its typical placement at the bottom of the lingual fossa were essential in establishing a comprehensive laboratory protocol [14].

Case presentation

A 21-year-old female presented with generalized spacing in both the upper and lower anterior teeth. The patient had a Class I molar and canine relationship with no skeletal discrepancies or major crowding. Her chief complaint was the unesthetic spacing between anterior teeth, and she opted for lingual orthodontic correction for aesthetic reasons.

The lingual surfaces of the maxillary and mandibular anterior teeth were evaluated for suitability for indirect bonding, considering variations in surface morphology and the depth of the lingual fossae. The simplified lingual indirect-bonding protocol described in this report was selected to enhance efficiency in bracket placement, ensure precise positioning, and address the patient’s aesthetic expectations.

All laboratory and clinical procedures - including bracket positioning, adhesive application, transfer tray fabrication, and the incorporation of incisal and occlusal windows - were performed using the workflow described in the Introduction section. The treatment objective was closure of anterior spacing while maintaining optimal tooth alignment and occlusal stability.

Methodology

Search Strategy

To enhance reproducibility and minimize selection bias, the literature search strategy was predefined and documented. Search terms included combinations of “lingual orthodontics”, “indirect bonding”, “transfer tray”, “HIRO technique”, “simplified lingual bonding”, “CAD/CAM”, and “tray accuracy”. Searches were conducted independently by the authors, and retrieved records were screened based on title and abstract relevance, followed by full-text assessment. Quantitative outcomes reported in the included studies - such as bonding accuracy, angular deviation, bond failure rates, chairside time, and cost estimates - were extracted only when explicitly stated in the original investigations. Studies lacking clear methodology or outcome definition were excluded from quantitative comparison and discussed qualitatively where relevant.

The methodology is described in the following sequential manner.

**Figure 1:**
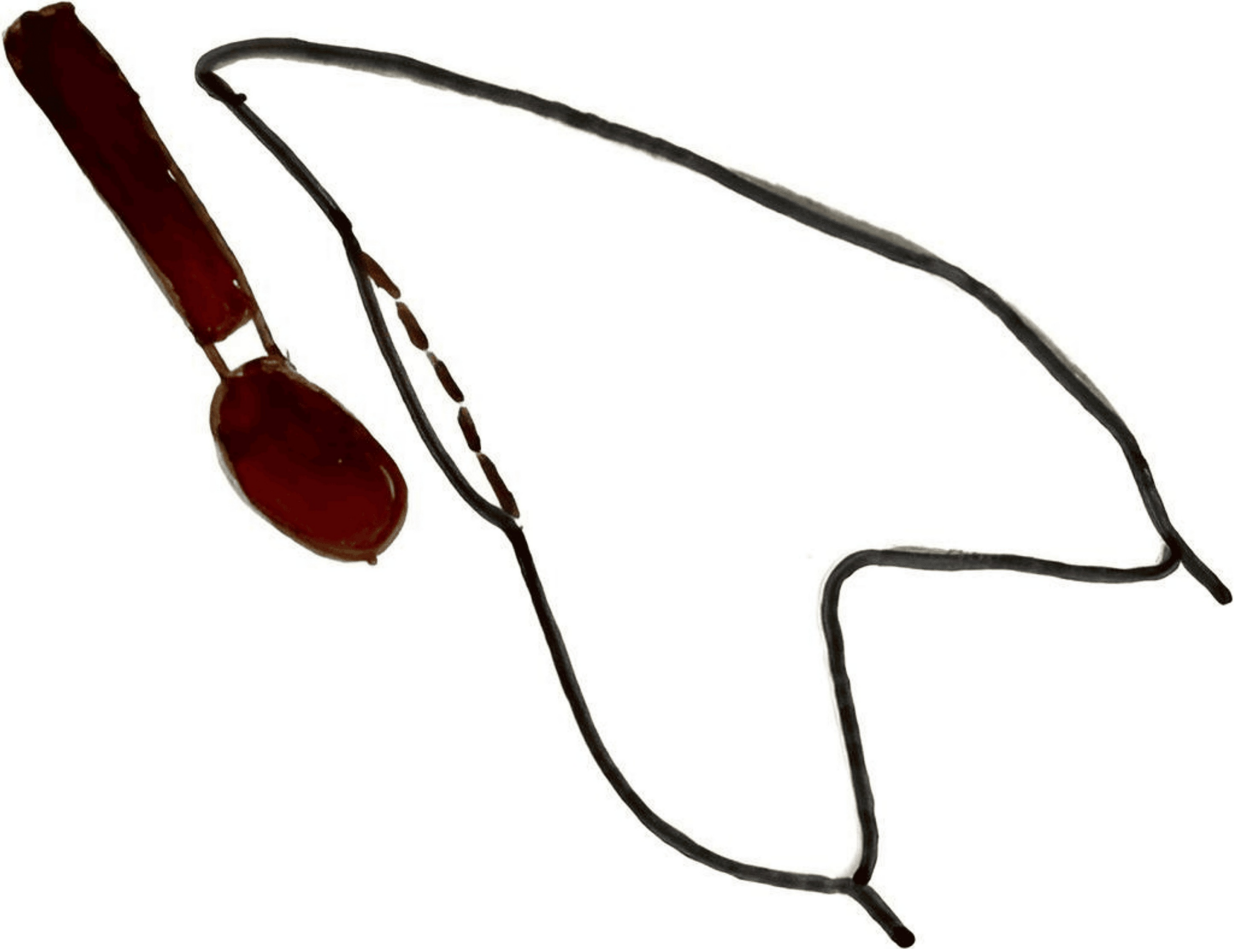
Preparation of unfavorable lingual tooth surface to facilitate bracket placement using the simplified lingual bonding system. This illustration demonstrates the selective enamel reduction performed on an irregular or unfavorable lingual surface using a rotary bur. The objective of this modification is to create a smooth, uniform bonding surface that improves bracket adaptation, enhances positional accuracy, and supports predictable bonding during the lingual orthodontic procedure. Image credit: Dr Nikita Agarwal

Mouth preparation: Check the lingual surface of anterior teeth and perform minor enamel adjustments if needed to ensure proper lingual bracket seating. Enamel adjustment is a part of orthodontics, even the lingual surfaces [15], but it should not be worn over occlusal-related areas, particularly where anterior guidance is received (Figure 1). If irregularities are present, carefully reduce the lingual surface using high-speed burs from the Shofu composite polishing kit (SHOFU, San Marcos, CA). Premature reduction can cause unintended iatrogenic wear in areas critical to anterior guidance.

Obtaining the working models*:* An impression was made using high-precision orthodontic alginate (Waldent FlexiPrint). Accurate alveolar ridges and teeth allowed the precise assembly of anatomical lingual brackets using the simplified lingual system. Models should be cast with defect-free type IV special plaster.

Determination of lingual bracket height: Bracket height is crucial in fixed orthodontics. In the simplified technique, STb brackets are placed directly on the lingual surfaces using placement pliers and tweezers, positioned 1.5-2 mm from the incisal edge of anterior teeth and at the center of the lingual crown height for posterior teeth (Table 1) [16]. For clarity, lingual bracket height is defined as the distance from the tooth's incisal margin to the bracket base's incisal margin. These measurements are indicated explicitly for normal arches without crown size and/or shape anomalies. The recommended measurements for the anterior teeth showed compatibility with the position of the brackets in the center of the clinical crown of the posterior teeth, which can be easily determined with the patient's models in hand.

**Table 1:** Bracket height recommendations of STb lingual system Ormco lingual brackets (vertical positioning in mm). Reference: [16]

Teeth	Central incisor	Lateral incisor	Canine	1^st^ Premolar	
Upper	2	1.5	2.5	Middle of the crown	
Lower	3	2	2.5	Middle of the crown	

**Figure 2:**
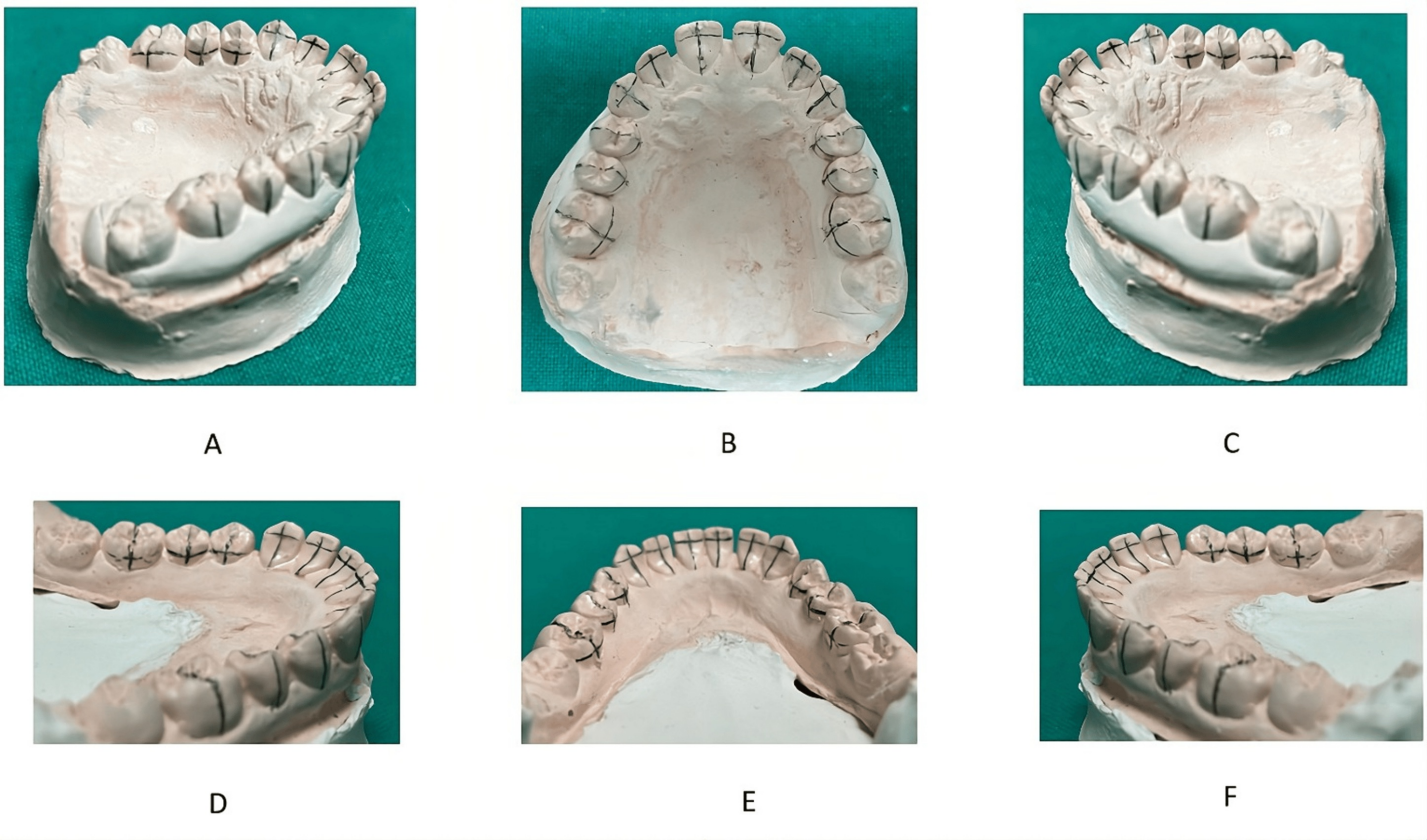
(A-C) Drawing of the guide lines for bracket positioning (as per the text) in the maxilla and (D-F) in the mandible. Original images from the study.

Drawing guidelines for bracket positioning: The following should be drawn with a graphite pencil on the models to serve as a guide for the positioning of lingual brackets (Figure 2): lingual cervical limit, incisal and occlusal limit, longitudinal axis of the crown on the lingual side, height of anterior brackets (determined by Table 1), and center of the lingual crown of posterior teeth.

Mounting brackets on the models: Apply a thin 1:1 (50:50) diluted layer of plaster insulator (Pyrax Cold Mould Seal) with water and allow it to dry. Fill bracket slots with wax to block bonding material. Then, attach brackets to the models using light-curing resin (Transbond™ XT, 3M Unitek) along the marked guide lines.

**Figure 3:**
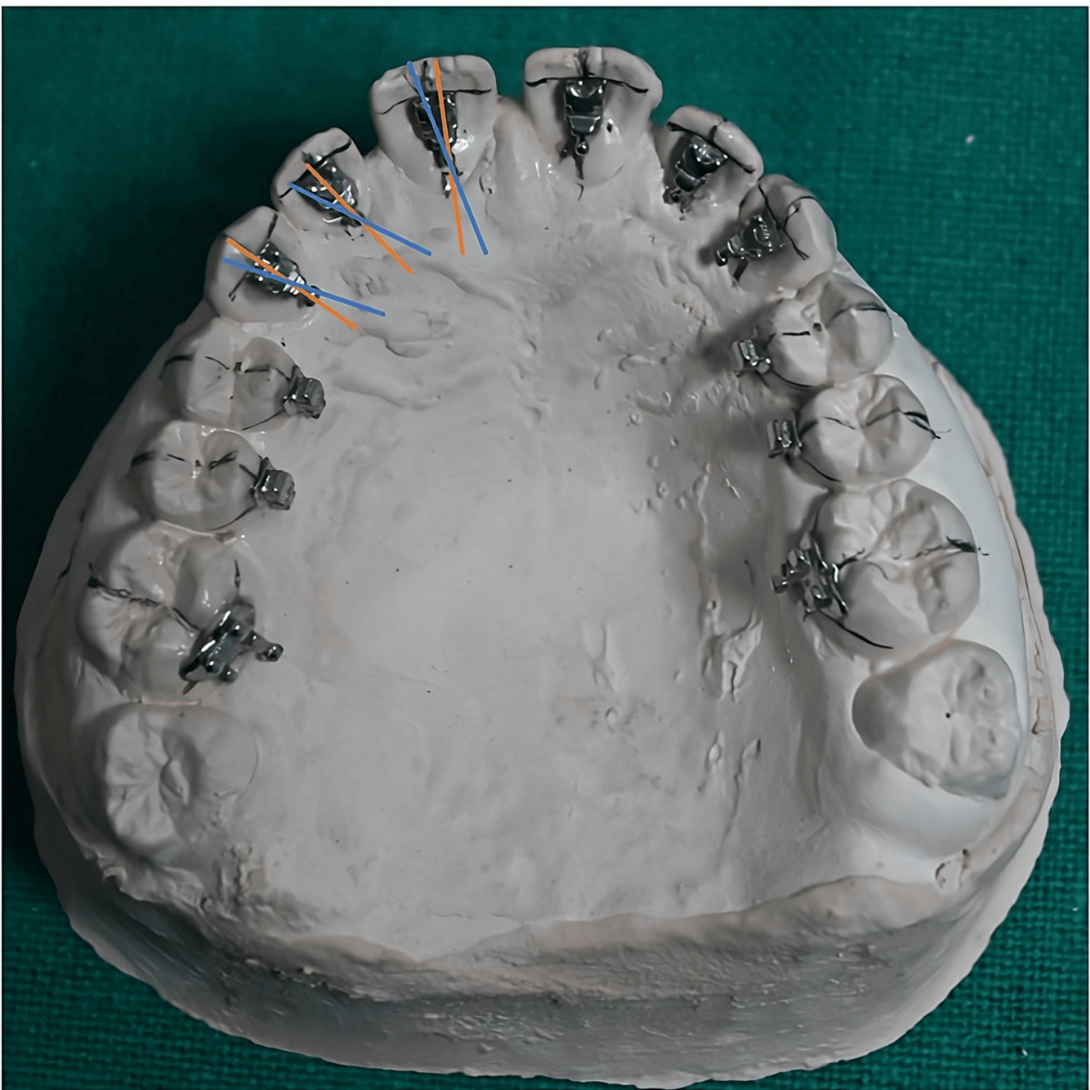
Position of anterior teeth brackets by the simplified lingual bonding technique. Original image from the study. The brackets of the incisors and canine are placed according to the guide lines drawn on the model where red lines depicted long axis of teeth and blue line depicted tip of the bracket.

Anterior teeth (upper and lower): The bracket base’s incisal edge should align with the crown height line, and bracket angulation is set by the slot’s tilt relative to the tooth’s long axis, following Ormco’s STb lingual bracket guidelines (Table 2 and Figure 3).

**Table 2:** Bracket angulation recommendations (mesiodistal tilt in degrees). Reference: [16]

Teeth	Recommended angulation
Central incisor	+5 to +7
Lateral incisor	+3 to +5
Canine	+8 to +10
Premolars	0 to +2
1^st^ Molar	0 to +3
2^ND^ Molar	0 to +2

**Figure 4:**
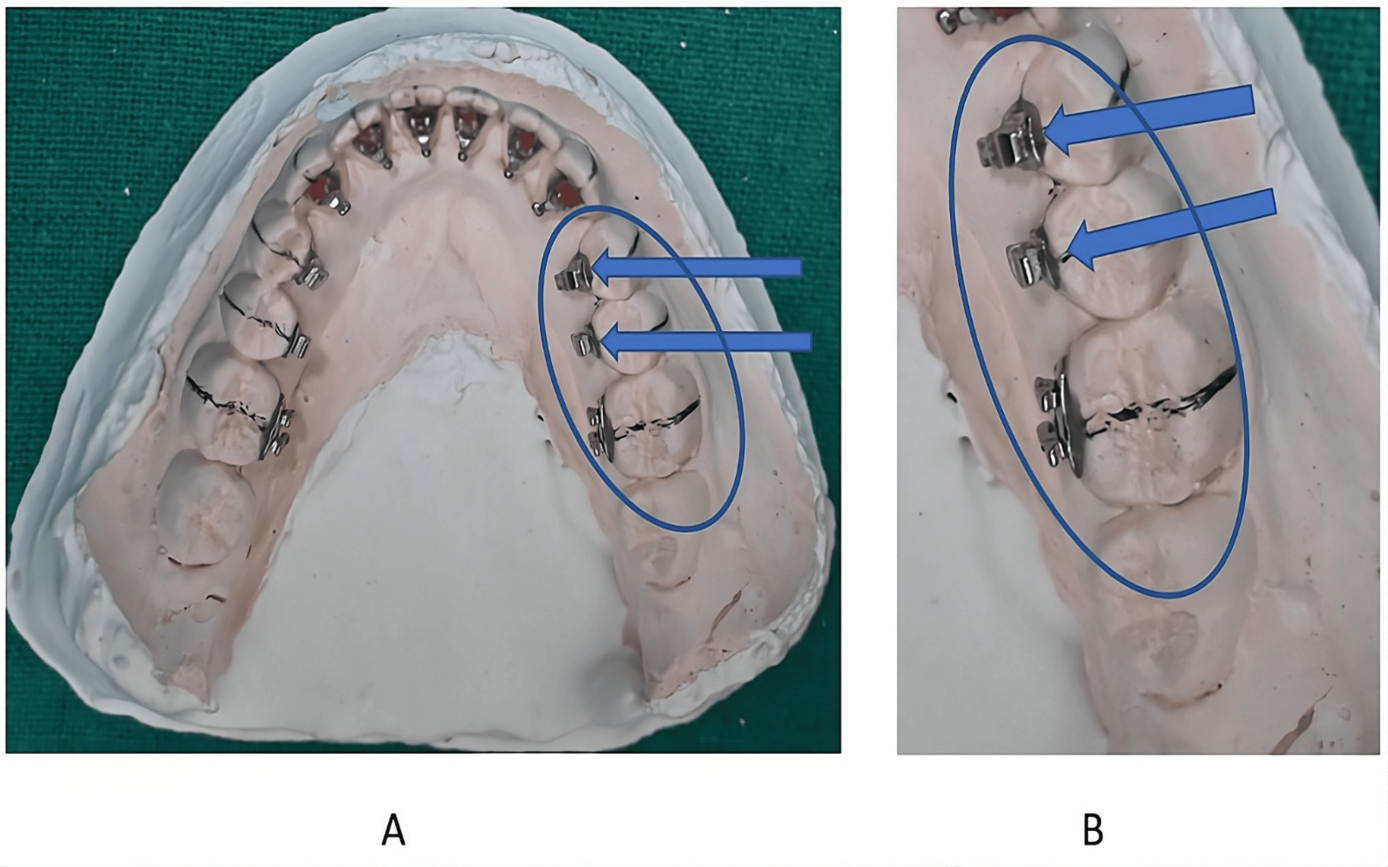
Position of the brackets on the lower premolars with a short lingual crown by the simplified lingual bonding system. Original images from the study. A layer of composite resin may be necessary on the occlusal surface, as shown in the model (A-B).

**Figure 5:**
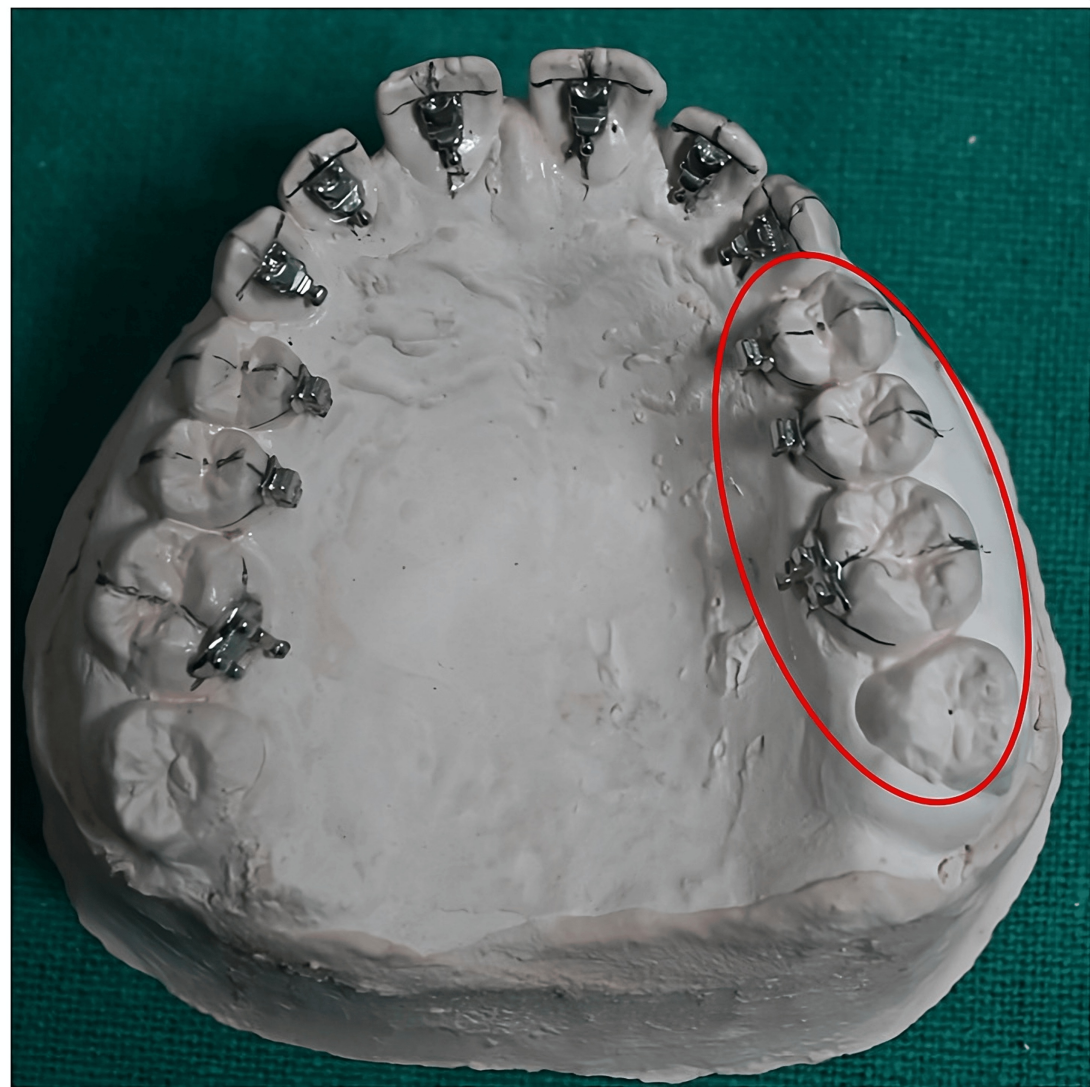
Position of the premolar brackets and molar tube on first molar by the simplified lingual system. Original image from the study.

Posterior teeth (upper and lower): During bracket placement on posterior teeth, a few points to be prioritized are the following: lower premolars frequently lack a lingual cusp. When this occurs, a resin layer should fill the space between the lingual tooth surface and the bracket base in its proper position ("over pad") (Figure 4). As shown in Figure 5, the position of brackets and molar tubes is denoted by bonding on the midpoint of the premolar and molar crown length when positioning the premolar brackets and molar tube.

Fabrication of the Transfer Trays

Lab procedure for the fabrication of the transfer tray, preparatory model preparation (as mentioned above), and design of the segmental tray: Three segmental trays (one anterior, two posterior) to enhance stability and moisture control with dimension as mentioned in palatal/lingual: 5-10 mm past cervical margin; labial extension: overlays the cervical third of teeth for clear vision; maxillary arch: up to 10 mm extension possible; and mandibular arch: restricted to 5 mm due to vestibular limitations.

Application of putty: Aquasil addition silicone putty (Aquasil TM, Soft Puuty/Regular set, Dentsply, Detrey). An equal amount of catalyst and base is mixed, and the anterior segment is canine-to-canine in the non-extraction and premolar-to-premolar in the extraction case. Use firm pressure to allow the total spread to interproximal and gingival regions without touching the incisal/occlusal surface.

**Figure 6:**
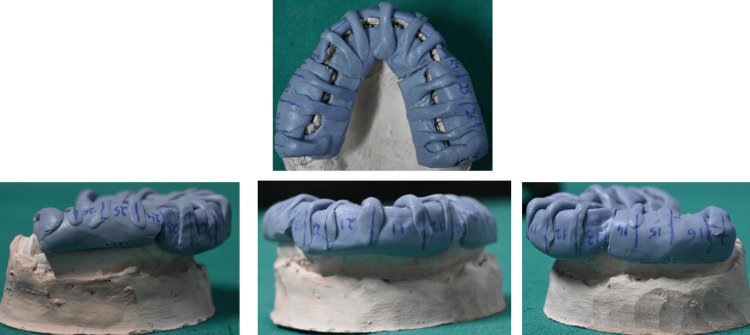
Anterior and posterior trays prepared (second PM extraction case). Original images from the study.

**Figure 7:**
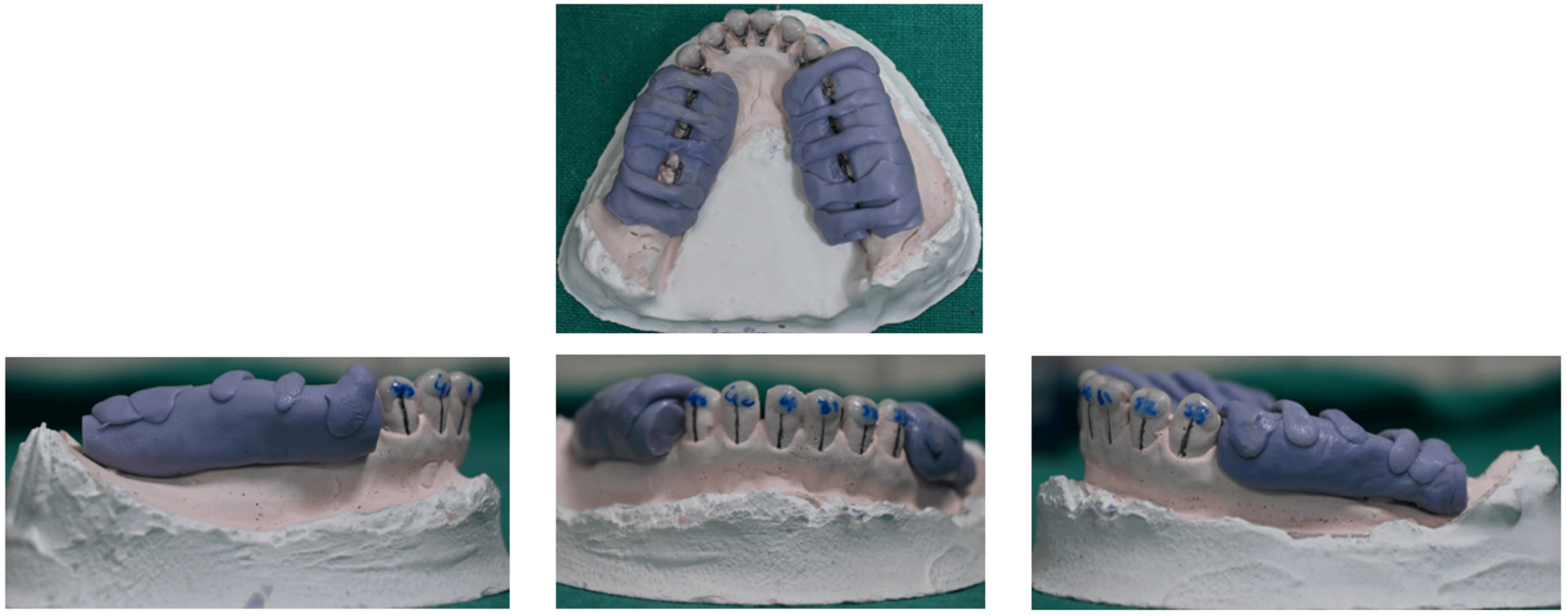
Individual resin-based transfer trays are made for the anterior teeth and posterior segmental trays prepared as followed for the maxilla. Original images from the study. Bracket bases are sandblasted.

Final tray assembly: A labial putty layer covers the incisal/occlusal surfaces to function as a vertical stop. Connectors are inserted between brackets, creating incisal/occlusal windows for light curing. A non-extraction case shows inadequate space for the mesial connectors of the posterior segmental tray. Conventionally, distal connectors of the anterior segmental tray are made first. The distal connectors of the posterior segmental trays are made after the removal of the anterior segmental tray in non-extraction cases. Teeth numbered with a permanent marker on the tray. Note that the putty has completely covered the gingival and interbracket area of the bracket base, and the free occlusal window can be seen. Bracket bases are sandblasted (Figures 6-7). A final thin putty layer is added for reinforcement and stiffening.

Tray finishing and removal: The tray design is refined with a BP blade if needed. Tooth numbering is marked on the trays for easy identification. The model is soaked in water (10-15 minutes) to soften the resin separator before carefully removing the transfer trays. Composite pads are sandblasted and alcohol-cleaned, making them ready for bonding.

Bonding process: (1) Isolation and surface preparation): The tooth surfaces are dried, cleaned, and isolated with absorbent rolls. Composite primer (Transbond ™ XT) is applied to both the tooth surface and bracket base. (2) Tray adaptation and curing: The transfer tray is placed and secured using labial and lingual finger pressure. Optimal tray adaptation ensures accurate bracket positioning. Brackets are light-cured at the occlusal windows for the desired period.

**Figure 8:**
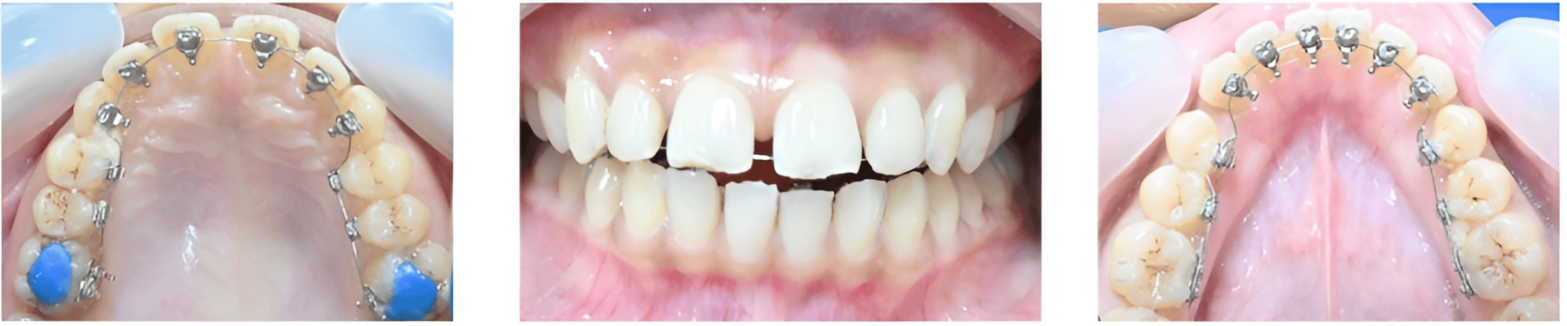
Lingual bracket bonded intraorally in the patient's mouth with posterior occlusal disocclusion by a blue bite. Original images from the study. Written informed consent for publication of these intraoral images was obtained from the patient.

Tray removal and final adjustments: The labial strip is drawn palatally/lingually, rupturing the connectors. A probe is employed to release the putty tray from the brackets. Do not pull the occlusal, as the well-fitting putty resists removal. Incisal/occlusal flash is eliminated using a 12-bladed carbide bur. Posterior occlusal lifts ("build-ups"): The occlusal lifts are made of composite resin in a non-tooth-colored shade (blue or white) to ensure easy identification during removal. The resin areas forming the "build-ups" are placed on the functional cusps of the upper first molars in the patient’s mouth (Figure 8). Archwire ligated to finalize the bonding process.

## Review

Discussion

Relevance of the Simplified Lingual Bonding System to the Present Case

The simplified lingual bonding system used in this case reflects a growing shift in orthodontic laboratory procedure. The technique was applied successfully in a 21-year-old female patient with a Class I molar and canine relationship and generalized spacing in both arches, and its application illustrates clinical advantages described throughout the literature, including improved adhesive control, enhanced curing access, and efficient tray removal.

Objectives of the Review

The objective of this review is to evaluate the efficiency and precision of simplified lingual indirect bonding systems with particular emphasis on laboratory workflows, transfer tray design, and bracket positioning accuracy. The review aims to compare simplified techniques with fully customized and HIRO-based approaches, synthesizing available evidence on clinical performance, procedural efficiency, and cost-benefit considerations.

Search Strategy

Electronic searches were performed using combinations of keywords related to lingual orthodontics, indirect bonding, transfer tray design, CAD/CAM workflows, and the HIRO technique.

Inclusion and Exclusion Criteria

In vitro studies, clinical studies, systematic reviews, and relevant technical reports were included. Studies unrelated to lingual orthodontics or indirect bonding accuracy were excluded.

Literature Review and Comparative Analysis

Over the past two decades, lingual orthodontics has seen significant advancements in bonding methodologies. Indirect bonding has emerged as the gold standard because it enhances bracket placement accuracy while reducing chairside time. Wiechmann’s research [17] on customized lingual brackets revolutionized the field, leading to the development of the Incognito system, which remains one of the most precise approaches to date. Although existing techniques provide robust stability and precision, making them reliable for complex cases, they can be time-consuming and prone to adhesive-related issues. Various studies, including those by Wiechmann et al. [17] and Hong [18], have outlined the complexities associated with lingual orthodontics, such as anatomical constraints, restricted visibility, and patient comfort. Keating et al. [19] and Nguyen [2] highlighted the need for enhanced workflows in lingual orthodontics, proposing simplified and precise tray designs for successful indirect bonding, supporting innovations such as windowed trays to optimize outcomes. Nguyen [20] noted that tray designs improve access and curing efficiency by enhancing clinical results in digital workflows. While specific studies on incisal and occlusal window trays are scarce, Sondhi [21] explored partial tray designs in labial orthodontics, noting benefits in adhesive control and curing access. The simplified technique with incisal and occlusal window transfer trays enhances workflow and patient experience by improving adhesive management, curing efficiency, and tray removal. This approach excels in scenarios where accessibility and efficiency are critical, marking it as a noteworthy advancement in lingual orthodontics. Key differences between the existing lingual bonding techniques and the simplified technique with incisal and occlusal window [17,19,21] (Table 3). Niu et al. [22] evaluated the different transfer tray designs used in lingual orthodontics. They found that transfer trays with incisal and occlusal windows reduced bonding failures and excess adhesive compared to full-coverage trays. These windows improved visualization and bracket positioning accuracy, though they slightly decreased tray stability, which can be managed with meticulous handling. Hiro [23] described a simplified lingual system where standard brackets are paired with customized composite bases, created on a model, and transferred via a tray, achieving results comparable to fully customized systems [23,24]. Here, we take a different approach by eliminating the need for an intermediate setup model while still utilizing a transfer tray system, thus reducing complexities. Table 4 presents the comparison between HIRO’s technique and a simplified technique [24,25]. Recent clinical adaptations of the HIRO technique further support its relevance in contemporary lingual orthodontics. Kara-Boulad et al. described a modified HIRO workflow in a clinical case report, demonstrating predictable bracket transfer and acceptable clinical efficiency using standardized brackets and customized composite pads rather than fully customized appliances [26]. This modified approach aligns conceptually with simplified lingual bonding philosophies, reinforcing the view that selective customization can achieve clinically satisfactory outcomes while reducing laboratory complexity.

**Table 3:** Key difference between the existing lingual bonding techniques and the simplified technique with incisal and occlusal window. References: [17,19,21]

Aspect	Existing Lingual Bonding Techniques	Simplified Technique with Incisal and Occlusal Window Tray
Tray Design	Uses full-coverage trays including incisal and occlusal surfaces	Tray with windows (cutouts) over the incisal edges and occlusal surfaces
Adhesive Management	Can trap excess adhesive (flash), complicating cleanup, especially on lingual surfaces.	Windows allows direct access to remove excess adhesive
Light Curing	Opaque trays may block light, requiring transparent materials or additional steps for proper curing	Windows enhance the light penetration, improving curing efficiency
Tray Removal	Extensive tooth contact can make tray removal difficult, risking bracket displacement	Reduced coverage via windows simplifies removal
Precision	Offers high precision	Maintains precision while simplifying the process, though windows may slightly reduce tray stability
Complexity and Time	Require more fabrication effort and chair time	Reduces fabrication complexity and procedure time
Patient Comfort	Bulkier trays may cause temporary discomfort during bonding	Less bulky trays with windows may improve comfort
Applicability	Suitable for a broad range of cases but may be overly complex for more straightforward treatments	This is ideal for cases prioritizing adhesive control and accessibility, especially in lingual orthodontics

**Table 4:** Comparison between Hiro’s technique and the simplified technique. References: [24,25]

Aspect	Hiro’s technique	Simplified Technique
Setup	Repositioned model	Malocclusion model
Placement	Archwire-guided	Manual, standardized
Complexity	High-skilled labor	Low, simple
Precision	High, suitable for complex cases	Moderate, suited for more straightforward cases
Equipment	Manual or digital tools	Essential tools only
Cost/Time	Costly and time-consuming	More economical and faster
Use Case	Best for complex malocclusions	Ideal for mild to moderate malocclusions
Trays	Rigid, reusable transfer trays	Flexible, simple trays

Compared to fully customized brackets, this approach simplifies the orthodontic process by eliminating the need for personalized brackets, reducing costs and production time. It maintains essential precision using customized bonding pads and the accuracy of the transfer tray. Lingual tooth anatomy varies significantly, affecting bracket design. This technique leverages the anatomical variance of the lingual fossa for bracket placement. Lombardo et al. [27] highlighted that anatomical irregularities necessitate individualized bracket positioning, often requiring compensatory bonding pads or customized bases. Three approaches address this: fully customized brackets, standard brackets with compensatory pads, and specific systems such as Hiro’s and STb lingual brackets [17,23,28]. In simplified lingual orthodontics, fully customized brackets are ideal for complex lingual anatomy, while standard brackets with pads, Hiro’s, and STb brackets are suited for simpler cases. Hiro’s brackets offer adaptability with larger bases, and STb brackets focus on compact design and light forces, enhancing efficiency in routine treatments. The adhesive protocol in simplified lingual orthodontics, using standard brackets with composite pads and windowed transfer trays, enhances bond strength, precision, and adhesive management. Studies such as Hiro [23] demonstrated that standard brackets with composite pads achieve bond strengths comparable to customized brackets. Das et al. [13] found that transfer trays with incisal and occlusal windows improve adhesive cleanup and curing efficiency, reducing bond failures by 30% compared to full-coverage trays. This confirms the effectiveness of the protocol, showing reduced bonding failures and enhanced clinical efficiency, making it ideal for routine lingual cases. Another key aspect of the simplified technique is its alternative approach to transfer trays, which emphasizes segmenting the tray into anterior and posterior sections. Although less rigidity may cause minor angular errors (~2° torque), segmented trays improve fit by adapting to irregular lingual anatomy, reducing bracket displacement in crowded cases [29]. Additionally, sectional bonding enhances moisture control by providing better isolation and strengthening bonds. Smaller segments reduce removal force, reducing bracket dislodgement risk by 15%. Studies by Alyammahi et al. [30] have highlighted the benefits of segmented trays, particularly in ensuring accurate seating in cases with severe malocclusion and crowding. Table 5 presents a comparison of the segmented and commercial trays [2,4].

**Table 5:** Comparison of the segmented and commercial trays. References: [2,4]

Aspect	Segmented	Commercial
Fabrication time	Take ~1-2 hours, 50% faster than commercial lab workflows	Takes days
Bonding Time	20-25 min/arch, 10% faster in crowded cases due to staged isolation	15-20 min/arch but less adaptable to moisture issues
Rebonding	20% more efficient for single brackets	Full-arch trays complicate rebonding
Cost	30-40% cheaper using standard materials	Expensive due to proprietary processes
Accuracy	Linear errors <0.1 mm, torque errors ~2°	Better angular accuracy (<1.5° errors) but less flexible

Clinical trials and retrospective studies provided strong evidence supporting the advantages of the simplified lingual indirect bonding technique. The simplified lingual indirect bonding technique demonstrates clear advantages over existing methods in accuracy, efficiency, reliability, and cost. It achieves high linear bonding precision (<0.1 mm) with minor angular deviations (~2°), aided by segmented trays with windows that enhance placement visibility [31]. While fully customized systems offer marginally better angular accuracy (<1.5°), they involve complex, time-consuming fabrication, and traditional full-arch trays are more prone to misalignment in crowded cases. Clinically, simplified trays reduced placement errors by 15% in mild-to-moderate crowding [2,30]. Regarding procedure time, the simplified method enables faster bonding and preparation due to in-house tray production and efficient adhesive cleanup, saving chair time [15,32]. This technique has lower bond failure rates (2-3%) due to better moisture control, outperforming traditional trays (5-7%) and aligning closely with customized systems [2]. Economically, the simplified approach significantly reduces costs (30-40%) using standard brackets and in-house processes, enhancing accessibility compared to expensive, proprietary customized systems [15]. El Sebaay et al. [29] demonstrated that segmented trays with incisal windows resulted in fewer placement errors and lower bond failure rates at six months compared to full-arch trays, due to improved adhesive control and operator ease. Das et al. [13] reported that the simplified method reduced bonding time and enhanced patient comfort by minimizing occlusal interference and simplifying tray removal. Nguyen [2] showed that this technique matched the accuracy of the Incognito system but was 30% cheaper and faster to produce, with fewer rebounds due to tray flexibility.

While the technique offers numerous advantages, it is not devoid of limitations. A significant drawback of this tray system is its insufficient rigidity for transferring individual tooth brackets, particularly in scenarios involving progressive bonding or rebonding of dislodged brackets. The limited space available for mesial and distal connectors, due to adjacent teeth having attached brackets, renders individual tooth occlusal window trays unreliable. Consequently, authors recommend utilizing Hiro’s transfer tray technique [24] or the Kommon base technique [33] for such cases. Further validation is necessary, particularly for cases involving severe crowding or significant variations in lingual anatomy. While our current study focuses on mild-to-moderate cases, there is a need for future studies to evaluate the performance of the simplified system in more complex clinical scenarios.

## Conclusions

Lingual orthodontics has evolved to meet the demand for discreet, efficient, and patient-friendly treatments. Traditional systems, while precise, often involve complex workflows, increased chair time, and higher costs. This case demonstrates that a simplified indirect lingual bonding technique incorporating incisal and occlusal windows segmented tray designs. This approach addresses adhesive cleanup, curing efficiency, and tray removal. Using standard brackets with customized composite pads and digital workflows, it achieves clinical accuracy comparable to existing systems while enhancing efficiency and reducing costs. Tray segmentation and anatomically based bonding improve adaptability, especially in crowded dentitions, and reduce bond failure rates and procedural time. Early clinical outcomes suggest this technique offers a compelling, accessible alternative in lingual orthodontics, particularly for mild to moderate cases, expanding high-quality treatment without compromising precision or patient experience.
